# Determinants of birth preparedness and complications readiness among mothers attending the antenatal care clinic at Katakwi General Hospital, Eastern Uganda: a cross-sectional study

**DOI:** 10.11604/pamj.2025.50.107.45514

**Published:** 2025-04-23

**Authors:** Benjamin Opus, Jane Francis Namatovu, Innocent Kabahena Besigye, Vincent Okuuny, Jude Tadeo Onyango

**Affiliations:** 1 Department of Family Medicine, School of Medicine, College of Health Sciences, Makerere University, Kampala, Uganda

**Keywords:** Determinants, mothers, pregnancy, antenatal care, delays, birth

## Abstract

**Introduction:**

Birth Preparedness and Complications Readiness (BPCR) is an essential component of antenatal care intended to reduce delays to access skilled care for birth and pregnancy/birth-related complications. Birth preparedness and complications readiness levels have been notably low, especially in sub-Saharan Africa and particularly in Uganda. This study established the level and determinants of birth preparedness and complications´ readiness among mothers attending the antenatal care clinic at Katakwi General Hospital.

**Methods:**

this was a cross-sectional study involving 378 mothers receiving antenatal care at Katakwi General Hospital between March and June 2024. Participants were interviewed using a structured questionnaire to record determinants and practices of birth preparedness and complications´ readiness. Three hundred and sixty-six questionnaires were analyzed, and 12 (3.2%) were dropped due to errors.

**Results:**

the overall level of birth preparedness and complication readiness was unsatisfactory. Antenatal care attendance was associated with better birth preparedness and complications readiness, while urban/semi-urban residence and history of miscarriage conversely had negative influences.

**Conclusion:**

the overall level of birth preparedness and complications´ readiness was unsatisfactory. Antenatal care attendance was associated with better birth preparedness and complications readiness while urban/semi-urban residence and history of miscarriage had negative effects. The health care providers should improve on mothers´ education to increase levels of birth preparedness and complications readiness. Educating/counselling mothers from urban/semi-urban areas and those with histories of miscarriages should be emphasized to encourage and improve practices of birth preparedness and complication readiness.

## Introduction

Maternal mortality is still a global public health challenge. According to estimates by the World Health Organization (WHO) in 2017, the global maternal mortality ratio was 211 deaths per 100,000 live births. Sub-Saharan Africa was estimated to have the highest maternal mortality ratio of 542 deaths per 100,000 live births, representing 66% of the global maternal deaths. The same report estimated Uganda´s maternal mortality ratio at 375 deaths per 100,000 live births [1,2]. Some reports have indicated a reduction in the institutional maternal mortality ratio (IMMR) in Uganda to 189/100,000 deliveries (194/100,000 live births), but this is still unacceptably high [3,4]. The majority of these deaths can be linked to delays in accessing skilled birth attendance. A systematic review for maternal deaths in a tertiary care hospital in Kampala, Uganda, revealed that 95% of maternal deaths had connections with delays in accessing care, and lack of BPCR was a significant contributory factor [5]. In the same study, 37% of the delays were attributed to the family level factors, 4% to transport issues at home, while 58% of the cases had delays at more than one point in the referral and care processes. The study also found that 84% of the deaths could have been prevented [5]. This underscores the need for mothers and their families to be prepared for birth. They should also be aware that complications commonly occur during pregnancy and birth, as noted in analyses by the WHO and studies in Ethiopia and Uganda [6-8].

Birth preparedness and complications readiness (BPCR) is an essential component of the antenatal care intended to reduce delays by the pregnant mother to access skilled services for birth and treatment of complications. These unnecessary delays can occur at home or at the health facility [9]. The BPCR concept itself has been demonstrated to promote skilled birth attendance and improve maternal and neonatal outcomes [10-14].

The WHO has designed specific interventions (elements) to enable the mother and her family to practice BPCR. These include knowing the desired place of birth, knowing the preferred birth attendant, knowing the closest health facility in case of emergency, having supplies and materials for birth, having a labor/birth companion, Identifying a supporter to look after the home and the other children while a mother is away during labor and birth and in case of any complications, identifying transport to the health facility for birth and in case of complications and identifying a compatible blood donor [15]. Different studies have used varying numbers of these practices to form composites for establishing overall levels of BPCR in the study populations. Despite its established role of promoting skilled birth attendance and improving maternal and neonatal outcomes, the levels of practice of the BPCR concept have remained low in many communities, as observed in studies in Ethiopia, Kenya, and Uganda [13,16-19].

Factors that may influence BPCR include: the mother´s sociodemographic characteristics, partner/family characteristics, the mother´s obstetric and gynecological characteristics, the mother´s knowledge of obstetric danger signs, and the mother´s knowledge about BPCR. A study in Ghana revealed that increasing maternal age was significantly associated with increasing levels of BPCR [20]. In a Tanzanian study, significant determinants of BPCR included the maternal level of education, the spouse´s employment, and antenatal care attendance [21]. In a narrative review of cross-sectional studies in sub-Saharan Africa, important determinants of BPCR were found to be maternal age and parity [22]. The knowledge of danger signs of pregnancy and birth has been significantly associated with the practice of BPCR, as noted in the studies in Ghana, Ethiopia, and Uganda [20,23,24]. Similarly, mothers´ knowledge about BPCR was found to be associated with better odds of being birth prepared and complications ready, as noted in studies in Ethiopia and Tanzania [21,25].

There was limited information about BPCR among mothers attending the antenatal care clinic at Katakwi General Hospital in Eastern Uganda. This study established the level and determinants of BPCR among mothers attending the ANC services at this hospital.

## Methods

**Study design, setting, and population:** this was a cross-sectional study conducted on mothers attending the antenatal care clinic at Katakwi General Hospital, a government health facility in Katakwi District, Eastern Uganda. Katakwi District had a projected population of 213,133 (financial year 2023/2024). The hospital, however, served a catchment population of 32,168. According to the Uganda MOH Health Management Information System Volume 1 Procedure Manual 2010, pregnant mothers constitute an estimated 0.5% of the catchment population [26]. Therefore, the number of pregnant mothers who were expected to attend this antenatal clinic for the financial year 2023/2024 (n) = 0.5/100 x 32,168 = 1608. The antenatal clinic was located in the maternity ward building and ran mainly on four days weekly: Monday, Tuesday, Wednesday, and Friday. The clinic was mainly run by midwives, while doctors were consulted when necessary. The average attendance per clinic day was 45 mothers. Pregnant mothers attending this clinic during the study period were included in this study. Mothers with unsound minds, enough to impede reasonable interviews, and those who declined to consent were excluded.

**Sample size estimation and sampling procedure:** the sample size was estimated using a formula for calculating sample sizes in cross-sectional studies.


$$N=\frac{Z{\left(1-\alpha /2\right)}^{2}P\left(1-P\right)}{d^{2}}$$

Where N is the required sample size and Z(1-α/2) is the standard normal value corresponding to the set level of confidence [27]. The standard normal of 1.96 was used for the 95% level of confidence; P is the estimated population proportion in previous studies.

The estimated proportion of 0.339 from a study in Ethiopia, where only 33.9% of mothers were prepared for birth, was used [16]; d is the level of precision/tolerable error. A five per cent level of precision was used based on the 0.339 estimated proportion.


$$\frac{1.96^{2}*0.339\left(1-0.339\right)}{0.05^{2}}=344$$

The calculated sample size, therefore, was 344. A top-up of 10% was considered to cater for possible errors in questionnaires. This brought the final sample size to 378. Systematic random sampling was used to select the individual participants to be interviewed. The list of pregnant mothers who came for antenatal care services on a particular day formed the sampling frame. The sampling interval was calculated using the formula; n (sampling interval) = K (population) ÷ N (sample size) [2]. Therefore, n (sampling interval) = 1608 (the calculated expected number of pregnant mothers in the catchment area) ÷ 378 (sample size) = 4.3. The sampling interval was therefore 4.

**Data collection:** data was collected by trained research assistants using a pretested structured questionnaire with socio-demographic characteristics, obstetric/gynecological characteristics, partner characteristics, knowledge of BPCR, and knowledge of danger signs as independent variables, and practice of BPCR as the dependent variable. This questionnaire was constructed on the basis of literature from previous studies and BPCR tools in a JHPIEGO manual (Monitoring BPCR, tools and Indicators for Maternal and Newborn Health) [28]. The questionnaire was structured to suit the local context. It was pretested at a nearby health facility´s (Aliakamer Health Centre III) ANC clinic, and some adjustments were made accordingly. The questionnaire was, however, not validated due to financial and time constraints.

The knowledge of BPCR was documented using a composite of 10 elements, and a mother who mentioned more than 5 elements was categorized as knowledgeable. The knowledge of danger signs was derived from a composite of 5 danger signs, and a mother who could spontaneously mention at least 3 danger signs was considered knowledgeable about the danger signs of pregnancy and birth. The practice of BPCR was documented using a composite of 9 elements, and a mother who practiced at least 5 elements was categorized as prepared for birth and ready for complications of pregnancy and birth. To mitigate potential facility bias, health care providers who served in the study facility (maternity) were not involved in the data collection exercise.

**Data analysis:** filled questionnaires were checked for accuracy and completeness before being considered for entry into an Excel sheet using EpiData client entry software, where characteristics were documented as binary variables with 1 and 2 codes. Three hundred and sixty-six (366) questionnaires qualified for entry, while 12 (3.2%) were dropped due to inaccuracies or incompleteness. Data in the Excel sheet was cleaned, and missing data, inaccurate, or double entries were corrected using the EpiData manager. Analysis was done using the EpiData analysis software. The bivariate analysis was done to determine crude ratios, and the independent variables whose P values were less than 0.05 were considered statistically significant. The independent variables whose P values were less than 0.5 were considered for multivariate analysis to adjust for confounders. Based on biological plausibility, age, gravidity, and modes of past delivery were also considered for multivariate analysis, though their P values were 0.5 and above in the bivariate model. Descriptive statistics, crude and adjusted odds ratios were then presented in tables, and further explanations done textually.

**Ethical considerations:** permission was obtained from the School of Medicine Research and Ethics Committee of Makerere University College of Health Sciences. The District Health Officer of Katakwi District and the Medical Superintendent of Katakwi General Hospital were informed about the study and the administrative clearance obtained. Consent was obtained from the mothers before inclusion as respondents.

## Results

**The mothers´ characteristics (sociodemographic, partner, and obstetric/gynecological):** the mothers´ characteristics are displayed in Table 1. Three hundred seventy-eight (378) mothers participated in the study, but 366 (96.8%) questionnaires were analyzed, while 12 (3.2%) were dropped due to inaccurate or incomplete data. Notably a half (50.5%) of the mothers were young (15-24 years of age). Three-quarters (74.9%) were from rural residence, while the other 25.1% hailed from urban or semi-urban communities. Three hundred twenty-two (88%) mothers reported to be either married or cohabiting, while the remaining 12% were either single, separated, or widowed.

**Table 1:** characteristics of participants selected from mothers attending the antenatal care clinic at Katakwi General Hospital during the study period (sociodemographic characteristics (N=366), partner characteristics (N=322), and obstetric and gynecological characteristics (N=366))

Variable	Variable’s characteristics	Frequency & (%)
**a) Sociodemographic characteristics (N=366)**		
Age	16-24 years	185 (50.5)
	25-45	181 (49.5)
Tribe	Ateso	346 (94.5)
	Kumam/Karimojong/others	20 (5.5)
Religion	Christian	363 (99.2)
	Muslim	3 (0.8)
Marital status	Married/cohabiting	322 (88)
	Single/divorced/separated	44 (12.0)
Residence	Rural	274 (74.9)
	Urban/semi-urban	92 (25.1)
Education	No formal/primary	259 (70.8)
	Secondary & above	107 (29.2)
Employment	Not employed	254 (69.4)
	Employed	112 (30.6)
Average monthly income	UGX < 100,000	295 (80.6)
	UGX ≥ 100,000	71 (19.4)
**b) Partner/family characteristics**		
Education status (n=322)	No formal/primary	172 (53.4)
	Secondary & above	150 (46.6)
Employment status (n=322)	Not employed	190 (59)
	Employed	132 (41)
Average monthly income (n=183)	UGX < 100,000	124 (67.8)
	UGX ≥ 100,000	59 (32.2)
**c) Obstetric and gynecological characteristics (N=366)**		
Gravida	1	99 (27.1)
	2-4	189 (51.6)
	≥ 5	78 (21.3)
Gestational age	< 37	347 (94.8)
	≥ 37	19 (5.2)
ANC visits attended	<4	226 (61.7)
	≥ 4	140 (38.3)
Estimated gestation age at first ANC visit	≥ 12	230 (62.8)
	< 12	136 (37.2)
H/O stillbirths	No	358 (97.8)
	Yes	8 (2.2)
H/O miscarriage	No	305 (83.3)
	Yes	61 (16.7)
Past modes of delivery (n=267)	Spontaneous vaginal deliveries	256 (95.9)
	C/section or assisted VD	11 (4.1)

ANC: antenatal clinic; H/O: history; VD: vaginal delivery

Regarding the obstetrical and gynecological characteristics of the mothers recruited in the study, half (51.6%) of them were carrying their second to fourth pregnancies, 27.1% were primigravidae, while the remaining 21.3% were carrying their fifth pregnancies and above. More than half (61.7%) had attended the ANC clinic less than 4 times. Also, more than half (62.8) had their first ANC visit late (at 12 weeks and above of their pregnancies). The majority (94.8%) of the respondents were less than 37 weeks into their pregnancies. Sixty-one respondents (16.7%) had ever experienced a miscarriage.

**Knowledge about BPCR, knowledge about danger signs, and practices/level of BPCR:** these can be viewed in Table 2. Less than a quarter (15.3%) of the respondents were considered knowledgeable about BPCR. These were respondents who were able to mention at least 3 of the 10 elements of BPCR. The most identified element was having a delivery kit, as mentioned by 87.4% of the mothers, followed by saving money for emergency or delivery (52.5%). Having a compatible blood donor(s) was mentioned by only 7 respondents (1.9%). The least known items as elements of BPCR were knowing a skilled birth attendant, knowing the health facility for birth, and identifying the health facility that operated 24 hours in case of emergency. These elements were mentioned by only 2 mothers (0.5%) each. Concerning sources of the information about BPCR (only provided in this text; not in table), of the 95% of the respondents who had reported that they had ever heard about the concept, their sources of information were; predominantly health care provider (91.7%), followed by radio (8%) and Community Health workers/Village Health Teams (VHTs) (7.4%). Print media, television, and the internet were largely unknown sources of information about BPCR, as reported by only one respondent (0.3%) each.

**Table 2:** knowledge about Birth Preparedness and Complications’ Readiness (BPCR), knowledge about danger signs and levels of BPCR practices among participants selected from mothers attending the antenatal care clinic at Katakwi General Hospital during the study period (N=366)

Mothers’ knowledge about BPCR (N=366)	
**Characteristic**	**Frequency (%)**
Not knowledgeable	310 (84.7)
Knowledgeable	56 (15.3)
**Mothers’ knowledge about individual elements of BPCR**	
**Element of BPCR**	**Frequency & (%)**
Identify a skilled birth attendant	2 (0.5)
Identify a health facility for birth	2 (0.5)
Identify a health facility that operates 24 hours in case of an emergency	2 (0.5)
Saving money for birth and in case of an emergency	192 (52.5)
Knowing danger signs	18 (4.9)
Arranging transport	43 (11.7)
Discuss the delivery plan with the partner	11 (3.0)
Arrange for a birth companion	21 (5.7)
Having a delivery kit (materials)	320 (87.4)
Having a compatible blood donor	7 (1.9)
**Mothers’ overall knowledge about danger signs (N=366)**	
**Characteristic**	**Frequency & (%)**
Not knowledgeable (knowing < 3 signs)	352 (96.2)
Knowledgeable (knowing ≥ 3 signs)	14 (3.8)
**Mothers’ knowledge about individual danger signs**	
**Danger signs of pregnancy and birth**	**Frequency & (%)**
Vaginal bleeding	157 (42.9)
Convulsions/loss of consciousness	11 (3.0)
Swollen hands/face or both	20 (5.5)
Drainage of waters before labor	25 (6.8)
Prolonged labor	46 (12.6)
**Overall prevalence/levels of BPCR practices (N=366)**	
**Characteristic**	**Frequency & (%)**
Unprepared	189 (51.6%)
Prepared	177 (48.4%)
**Mothers’ levels of practice for individual elements of BPCR**	
**BPCR component**	**Frequency & (%)**
Knowing a skilled birth attendant	231 (63.1)
Knowing a health facility for birth	233 (63.7)
Knowing a health facility that operates 24 hours in case of an emergency	217 (59.3)
Save money for birth & emergency in case of an emergency or labor	59 (16.1)
Arrange for transport	101 (27.6)
Arrange for a birth companion	151 (41.3)
Preparing for a delivery kit (materials)	221 (60.4)
Having a compatible blood donor	16 (4.4)
Discussing the delivery plan with partner/family	91 (24.9)

On the mothers´ knowledge of danger signs of pregnancy and birth, only 3.8% of the respondents were considered knowledgeable. These were respondents who were able to mention (unprompted responses) at least 3 of the five key danger signs of pregnancy and birth. The symptom that was spontaneously mentioned by most but less than a half (42.9%) of the respondents was vaginal bleeding. This was followed by prolonged labor (12.6%), drainage of waters before labor (6.8%), swollen hands and/or face (5.5%). Convulsions/loss of consciousness was mentioned by only 3% of the respondents.

This study sought to establish the levels of BPCR, and almost half (48.4%) of the respondents were considered prepared for birth and ready for its complications. These were the mothers who fulfilled (reported to practice) at least five out of nine elements of BPCR. Both unprompted and prompted responses were considered but with no double reporting to either category. The most practiced elements of BPCR were identifying a skilled birth attendant (63.4%) and identifying a health facility for birth (also 63.4%). Having a delivery kit (materials) was mentioned by 60.4% of the respondents. Also, more than half (59.3%) of the respondents knew a health facility that operated 24 hours in case of emergency. Less than half (27.6%) of the respondents had arranged for transport in case of emergency and labor. Less than a quarter (16.1%) had saved money. The least reported practice of BPCR was having a compatible blood donor (4.4%).

**Bivariate analysis:** at this bivariate level, the independent variables whose P values were less than 0.05 were considered statistically significant. The bivariate analysis of the sociodemographic and partner characteristics associated with BPCR (Table 3) found that marital status and mothers´ knowledge about BPCR were statistically significant. Mothers who were not married were less likely to be birth prepared than their married/cohabiting counterparts (OR 0.51, 95% CI 0.26, 0.98, P 0.046). Mothers who were considered knowledgeable about BPCR were almost twice more birth prepared and complications ready than their counterparts who were not knowledgeable (OR 1.97, 95% CI 1.11, 3.59, P 0.023). On the other hand, knowledge about danger signs was not significantly associated with BPCR.

**Table 3:** bivariate analysis of socio-demographic and partner characteristics associated with the levels of Birth Preparedness and Complications’ Readiness (BPCR) among participants selected from mothers attending the antenatal care clinic at Katakwi General Hospital during the study period (N=366 for sociodemographic characteristics, N=322 for partner characteristics except for partners’ average monthly income (N=183))

Variable	Variable Characteristics	Unprepared n=189, F & (%)	Prepared n=177 F & (%)	OR (95% CI)	P-value
Age	18-24	95 (50.3)	90 (50.8)	1	
	25-45	94 (49.7)	87 (49.2)	0.98 (0.65, 1.47)	0.911
Tribe	Ateso	177 (93.7)	169 (95.5)	1	
	Kumam/Karamojong/Others	12 (6.3)	8 (4.5)	0.70 (0.27, 1.73)	0.444
Marital status	Married/cohabiting	160 (84.7)	162 (91.5)	1	
	Single/divorced/Separated	29 (15.3)	15 (8.5)	0.51 (0.26, 0.98)	0.046
Residence	Rural	134 (70.9)	140 (79.1)	1	
	Semi-urban/urban	55 (29.1)	37 (20.9)	0.64 (0.40, 1.04)	0.072
Education status	No formal/primary	137 (72.5)	122 (68.9)	1	
	Secondary &above	52 (27.5)	55 (31.1)	1.19 (0.76, 1.87)	0.454
Regular employment	No	125 (66.1)	129 (72.9)	1	
	Yes	64 (33.9)	48 (27.1)	0.73 (0.46, 1.14)	0.162
Average monthly income	<100,00 UGSH	151 (79.9)	144 (81.4)	1	
	≥100,000 UGSH	38 (20.1)	33 (18.6)	0.91 (0.54, 1.53)	0.724
Knowledge about BPCR	No	168 (88.9)	142 (80.2)	1	
	Yes	21 (11.1)	35 (19.8)	1.97 (1.11, 3.59)	0.023
Knowledge about danger signs	No	185 (97.9)	167 (94.4)	1	
	Yes	4 (2.1)	10 (5.6)	2.77 (0.91, 10.25)	0.090
**Partner characteristics (N=322) except average monthly income (n=183)**					
Variable	Variable characteristics	Unprepared F & (%)	Prepared F & (%)	OR (95% CI)	P value
Education	≥ Secondary	84 (52.5)	88 (54.3)	1	
	No formal/primary	76 (47.5)	74 (45.7)	0.93 (0.60, 1.44)	0.743
Regular employment	Yes	99 (61.9)	91 (56.2)	1	
	No	61 (38.1)	71 (43.8)	1.27 (0,81, 1.98)	0.299
Average monthly income (n=183) *	≥100,000 UGSH	61 (70.1)	63 (65.6)	1	
	< 100,000 UGSH	26 (29.9)	33 (34.4)	1.23 (0.66, 2.30)	0.517

* Mothers who were able to estimate their partners’ monthly income

In the bivariate analysis of the obstetric/gynecological characteristics (Table 4), gestational age and ANC attendance were found to be significantly associated with BPCR. Mothers who were 37 weeks and above were more than 3 times likely to be birth prepared and complications ready (OR 3.16, 95% CI 1.18, 9.95, P 0.031). Mothers who had attended the ANC clinic four times and above were more than twice as likely to be birth prepared and complications ready than their counterparts who had attended less than 4 times (OR 2.36, 95% CI 1.54, 3.65, P <0.001).

**Table 4:** bivariate analysis of obstetric/gynecological characteristics associated with levels of BPCR among participants selected from mothers attending the antenatal care clinic at Katakwi General Hospital during the study period (N=366), except for modes of past deliveries (N=267)

Variable	Variable characteristics	Unprepared n = (189) F & (%)	Prepared (n =177) F & (%)	OR (95% CI)	P-value
Gravida	1	54 (28.6)	45 (25.4)	1	
	2-4	101 (53.4)	88 (49.7)	1.05 (0.64, 1.71)	0.858
	≥5	34 (18.0)	44 (24.9)	1.55 (0.86, 2.84)	0.149
Gestational age	<37	184 (97.4)	163 (92.1)	1	
	≥37	5 (2.6)	14 (7.9)	3.16 (1.18, 9.95)	0.031
Ever had a stillbirth	No	182 (96.3)	176 (99.4)	1	
	Yes	7 (3.7)	1 (0.6)	6.77 (1.19, 127.20)	0.075
Ever had a miscarriage	No	153 (81.0)	152 (85.9)	1	
	Yes	36 (19.0)	25 (14.1)	0.70 (0.40, 1.22)	0,208
ANC visits	<4	135 (71.4)	91 (51.4)	1)	
	≥4	54 (28.6)	86 (48.6)	2.36 (1.54, 3.65)	<0.001
Gestational age at first ANC visit	≥12	114 (60.3)	116 (65.5)	1	
	<12	75 (39.7)	61 (34.5)	0.80 (0.52, 1.22)	0.302
ANC visits planned	<8	92 (48.7)	97 (54,8)	1	
	≥8	97 (51.3)	80 (45.2)	0.78 (0.52, 1.18)	0.242
Modes of past deliveries (n=267)	Characteristics	n=135	n=132		
	SVD	130 (96.3)	126 (95.5)	1	
	C/S or AVD	5 (3.7)	6 (4.5)	1.24 (0.36, 4.39)	0.730

ANC: antenatal clinic; SVD; spontaneous vaginal delivery; C/S: cesarean section; AVD: assisted vaginal delivery

Largely, partner/family characteristics were not associated with BPCR at both bivariate and multivariate levels (at the P value of <0.05).

**Multivariate analysis:** from the bivariate analysis, dependent variables whose P values were less than 0.5 and those considered for their biological plausibility (age, gravidity, and modes of past delivery) were considered for multivariate analysis. The multivariate analysis of the sociodemographic/partner and obstetric/gynecological characteristics (Table 5) revealed that residence, history of miscarriage, and ANC attendance were significantly associated with BPCR. Mothers who lived in semi-urban/urban settings were almost twice less likely to be birth prepared and complications ready than their counterparts who hailed from the rural areas (AOR 0.59, 95% CI 0.35, 0.98, P 0.046). Mothers who had ever had a miscarriage were also almost twice less likely to be birth prepared and complications ready (AOR: 0.47, 95% CI: 0.22, 0.98, P 0.048). The ANC attendance that was associated with BPCR at the bivariate level was still statistically significant at the multivariate level. Mothers who had attended antenatal care 4 times or above were more than three times as prepared and complications ready than their counterparts who had attended less than 4 times (AOR 3.12, 95% CI: 0.79, 5.56, P <0.001).

**Table 5:** logistic regression of sociodemographic, partner, and obstetric/gynecological factors associated with Birth Preparedness and Complications’ Readiness (BPCR) among participants selected from mothers attending the antenatal care clinic at Katakwi General Hospital during the study period

Variable	Variable characteristics	AOR	95% CI	P value
**Demographic factors**				
Age	18-24	1.00		
	25-45	0.98	0.63,1.51	0.914
Residence	Rural	1.00		
	Semi-urban/urban	0.59	0.35, 0.98	0.046
Education	No formal/primary	1.00		
	≥ secondary	1.35	0.82, 2.21	0.237
Regular employment	No	1.00		
	Yes	0.8	0.49, 1.30	0.375
Knowledgeable about BPCR?	No	1.00		
	Yes	1.84	0.99, 3.43	0.052
Knowledgeable about danger signs?	No	1.00		
	Yes	2.21	0.68, 8.59	0.208
**Partner characteristics**				
Education	≥ secondary	1.00		
	< secondary	0.84	0.52, 1.34	0.461
Regular employment	Yes	1.00		
	No	1.35	0.84, 2.18	0.219
**Obstetric/gynecological characteristics**				
Gravida	1	1.00		
	2-4	1.39	0.75, 2.49	0.312
	≥5	1.78	0.97, 3.32	0.064
Gestation age (weeks)	<37	1.00		
	≥37	2.05	0.55, 10.08	0.320
H/O stillbirth	No	1.00		
	Yes	0.15	0.01, 0.96	0.091
H/O miscarriage	No	1.00		
	Yes	0.47	0.22, 0.98	0.048
ANC visits attended	<4	1.00		
	≥4	3.12	0.79, 5.56	<0.001
Gestational age at first ANC visit (weeks)	≥12	1.00		
	<12	0.77	0.42, 1.42	0.401
ANC visits planned	<8	1.00		
	≥8	0.58	0.32, 1.05	0.076
Modes of past deliveries	SVD	1.00		
	C/section or assisted VD	1.89	0.47, 8.22	0.374

H/O: history; ANC: antenatal clinic; VD: vaginal delivery; SVD: spontaneous vaginal delivery

Marital status, gestational age, and knowledge about BPCR that were associated with BPCR at the bivariate level lost their statistical significance at the multivariate level.

## Discussion

This study established the level and determinants of BPCR among mothers attending the antenatal care clinic at Katakwi General Hospital. The level of BPCR practices was not satisfactory (48.4%). This level of BPCR is similar to that of the Ethiopian study, but a higher level had been encountered in the Tanzanian study [21,29]. However, lower levels than we found had been reported in studies in Bangladesh, Ghana, Rwanda, and Uganda [20,30-32]. The variation in the composition of the BPCR elements considered by different studies could have partially contributed to the wide differences in levels of BPCR. For example, while our study considered nine elements of BPCR, one Ethiopian study used twelve and another eleven elements [16,33]. Two studies in Ghana and Tanzania used five elements each, yet one in Uganda used as few as three [20,21,31]. The differences in study populations could also have partly impacted the levels of BPCR. Our study was among those that investigated these concepts in prenatal mothers, while other studies were done on postnatal mothers at different periods after birth. The most reported practices of BPCR by mothers recruited in this study were knowing a skilled birth attendant, knowing the health facility for birth, preparing a delivery kit/material, and knowing a health facility for emergencies. These findings are similar to those encountered in studies in Ghana and Tanzania [20,21]. In our study, the least reported practice of BPCR was having a compatible blood donor, and this was in agreement with findings in one study in Northern Uganda and another in Tanzania [19,20]. For the case of Uganda, mothers may not have the incentive to have a blood donor as all blood is collected mainly from public drives. The poor scores encountered in our study for some key elements of BPCR, like saving money, arranging for transport, and having compatible blood donors, could also be due to the poor content of the mother´s education in the ANC clinic, where much emphasis is placed on preparing materials for delivery. This is supported by the fact that this element (preparing delivery kit/materials) was among the most reported practices of BPCR in this study.

It is important that mothers be equipped with knowledge about BPCR, as this could translate into improved BPCR practices. The knowledge levels in our study were low. The most reported element in this study was having a delivery kit. Respondents could hardly mention skilled birth attendants, a health facility for birth, a health facility for emergency, and having a compatible blood donor as elements of BPCR. This points to poor content of ANC education for mothers, as emphasis may have been put on having a delivery kit. However, at the multivariate level, the knowledge about BPCR was not statistically significant.

Mothers also need to have a good knowledge of the danger signs of pregnancy and birth in anticipation of complications, which are often foreseeable. This would motivate mothers to seek skilled birth attendance and early care in case of emergency. In this study, mothers had very low levels of knowledge about danger signs, far much lower than levels found in studies in Ghana, Tanzania, and Uganda [17,20,21]. We considered five key danger signs that are common to pregnancy and/or labor and birth; vaginal bleeding, convulsions/loss of consciousness, swollen hands/face, drainage of waters before labor and prolonged labor. The most mentioned danger sign was vaginal bleeding as was similarly found in the study in Northern Uganda [19]. The least mentioned danger sign in this study was convulsions/loss of consciousness. The low levels of knowledge about danger signs could be due to poor quality of information/education for mothers attending the ANC clinics and inadequate or poor content of the community health workers´ programs regarding pregnancy and birth. This is supported by the fact that most mothers had reported that the health care providers and the community health workers were key sources of their information about BPCR. As a determinant of BPCR in this study, however, knowledge of danger signs was not statistically significant.

Determinants that were significantly associated with BPCR at the multivariate level were residence, ANC attendance, and history of having ever had a miscarriage. The mothers who resided in semi-urban/urban areas were less prepared for birth and ready for complications. Most studies, such as in Ethiopia and Nigeria, had found better odds of BPCR among urban dwellers [34,35]. The poor odds of BPCR among urban dwellers noted in our study could be due to being in close proximity to the health facility, which could have resulted in some complacency with reproductive health matters. Mothers who are residents in rural areas, on the other hand, may have better community mobilization/education and also be keen about their reproductive health as they are farther from the health facility. This supposition accedes to findings in a study in Thailand where mothers who lived farther from the health facility were better prepared for birth and complications ready [36]. Our study revealed higher odds of BPCR among mothers with an increasing number of ANC visits. This agrees with findings of studies in Tanzania and Kenya [21,37]. This supposes that mothers get more exposed to information with more ANC attendance and underscores the need for mothers to start their ANC early, and BPCR messages to be emphasized during all subsequent visits. A mother will also tend to be more prepared as she approaches her date of birth. In the Ghanaian study, however, increasing attendance of ANC did not improve the odds of being birth prepared and complications ready [20]. In our study, a history of having ever had a miscarriage was significantly associated with lower odds of being birth prepared and complications ready. This could be due to another pregnancy happening too soon before the mother is prepared. It could also be due to demotivation among these mothers from the difficult obstetric experiences, coupled with inadequate support in the maternity care processes. Studies in Ethiopia had, however, revealed higher odds of BPCR among mothers with unpleasant obstetric experiences such as the history of stillbirths [23,38].

In this study, there was a high proportion of young mothers, an indicator of early marriages, early sexual debut, unprotected sex, and lack of contraception among this age group. This needs further review. Nevertheless, in this study, maternal age had no significant association with BPCR.

**Strengths of this study:** according to published information, this study was one of its kind in Katakwi District. There was also no accessible published study on BPCR in the Teso sub-region in Eastern Uganda, where Katakwi is located. This study used an interviewer-administered questionnaire approach of data collection, which improves the respondents´ understanding of the questions and minimizes response biases.

**Weakness of the study:** two concepts (birth preparedness and complications readiness) were studied together as one dependent variable, yet the study was conducted on prenatal mothers who were at different gestational ages. This may have introduced a response bias, especially from mothers in their early gestational ages.

The study was conducted in one health facility, and this affects the representativeness of the study results. Involving more health facilities at different levels could have produced better generalizable results.

## Conclusion

The overall level of BPCR established by this study was not satisfactory. The District Health Office should support the health care providers to repackage the BPCR messages with emphasis on elements that were poorly understood and practiced by mothers in this study. The determinants associated with BPCR in this study were: residence, antenatal care attendance, and history of miscarriage. Antenatal care attendance was found to positively influence BPCR. Birth Preparedness and Complications´ Readiness (BPCR) messages should therefore be emphasized during the early ANC visits so that mothers timely appreciate the concepts. Health education and individual counselling on BPCR should then be continued during all subsequent ANC visits to help mothers consolidate their practices. Semi-urban/urban residence and history of miscarriage were negatively associated with BPCR. The Health Care Providers should offer more targeted counselling and education support to mothers from urban/semi-urban areas and those with histories of miscarriages and other poor obstetric histories.

### What is known about this topic

- Increasing maternal age is associated with better odds of BPCR practice;
- Increasing ANC attendance positively influences levels of BPCR;
- Mother’s knowledge about danger signs of pregnancy increases the odds of being birth prepared and complications ready.

### What this study adds

- Being a resident in urban/semi-urban areas is associated with lower odds of BPCR practice;
- Mothers with histories of miscarriages can be less prepared for birth and ready for complications of pregnancy and birth.
